# Citation self-awareness for a fairer academic publishing landscape

**DOI:** 10.1093/biosci/biag028

**Published:** 2026-04-09

**Authors:** Miriam Beck, Pavanee Annasawmy, Deborah Birre, Michela Busana, Nicolas Casajus, Camille Coux, Clara Marino, Nicolas Mouquet, Lisa Nicvert, Brunno F Oliveira, Cathleen Petit-Cailleux, Axelle Tortosa, Mithila Unkule, Chloé Vagnon, Devi Veytia

**Affiliations:** FRB-CESAB, Montpellier 34070, France; FRB-CESAB, Montpellier 34070, France; FRB-CESAB, Montpellier 34070, France; FRB-CESAB, Montpellier 34070, France; FRB-CESAB, Montpellier 34070, France; FRB-CESAB, Montpellier 34070, France; FRB-CESAB, Montpellier 34070, France; FRB-CESAB, Montpellier 34070, France; MARBEC, University of Montpellier, CNRS, Ifremer, IRD, Montpellier 34095, France; University of Montpellier, Montpellier 34090, France; FRB-CESAB, Montpellier 34070, France; FRB-CESAB, Montpellier 34070, France; INRAE, LESSEM, St-Martin-d’Hères 38402, France; FRB-CESAB, Montpellier 34070, France; Université de Toulouse, INRAE, DYNAFOR, Castanet-Tolosan, 31326, France; FRB-CESAB, Montpellier 34070, France; French Foundation for Biodiversity Research (FRB), Paris, 75005, France; FRB-CESAB, Montpellier 34070, France; FRB-CESAB, Montpellier 34070, France

**Keywords:** academic publishing, citation bias, citation practices, publishing ethics, shared profit publishing

## Asymmetries in the academic publishing landscape

Academic journals are essential for the scientific process by validating research through rigorous peer review, disseminating knowledge, and preserving scientific record. As gatekeepers of science, journals, and their governing bodies carry significant responsibility toward the long-term interests of the research community. The diverse landscape of academic publishing encompasses journals that differ in disciplinary scope, editorial selectivity, audience, ownership structures, and modes of access. In addition, journals span a continuum of business models: one end comprises for-profit (FP) journals, which primarily return revenues to shareholders; the other harbors non-profit (NP) journals, which reinvest any potential revenues into the academic ecosystem.

**Figure 1.**
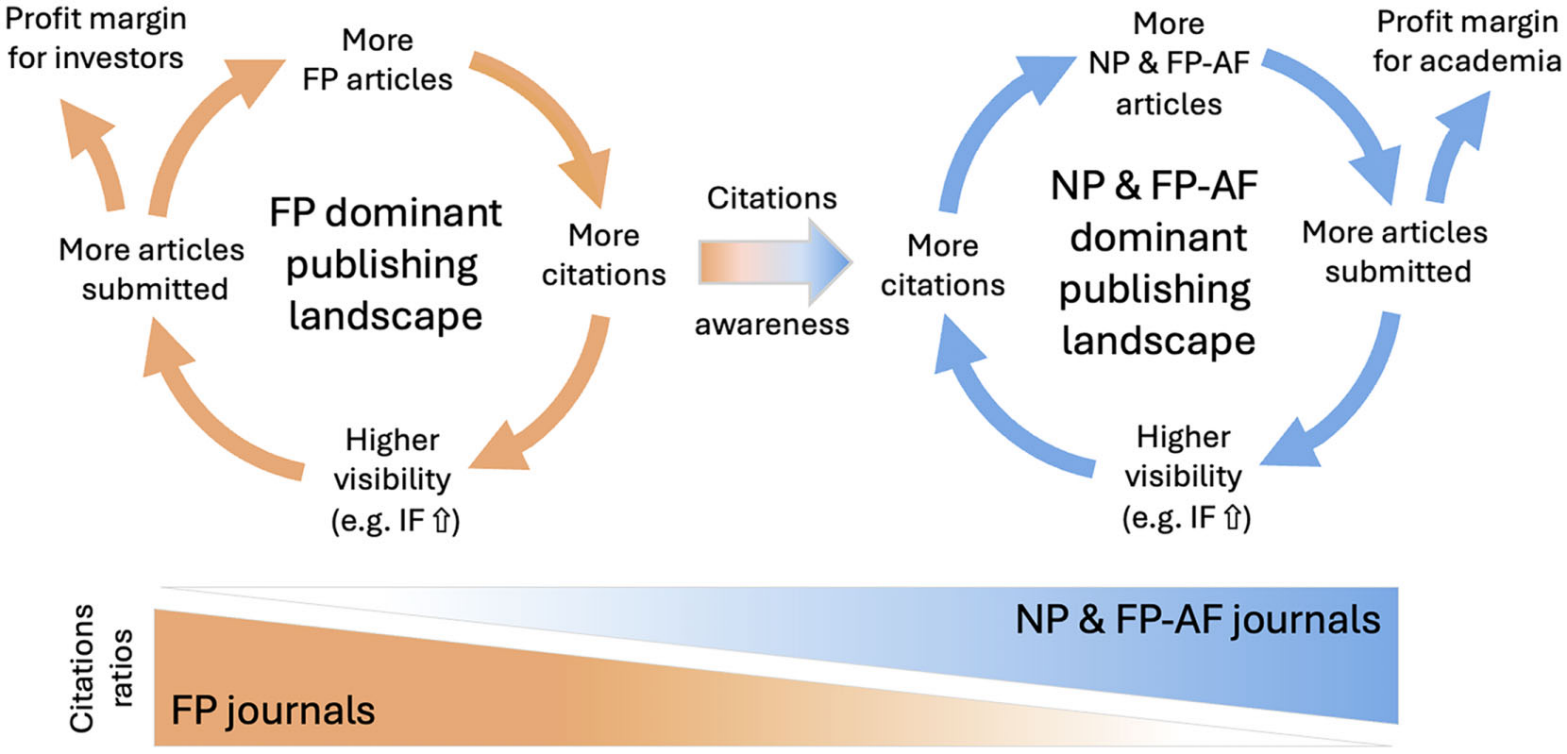
Self-reinforcing dynamic of publishing landscape. Journals are shown to operate within a positive feedback loop in which higher visibility, often mediated through journal-level metrics such as the impact factor (IF), attracts more submissions, leading to increased publication volume, citations, and financial returns to investors (FP, for-profit journals; FP-AF, for-profit academia-friendly journals; NP, non-profit journals). This dynamic contributes to the growing dominance of FP journals within the scholarly publishing ecosystem given their numerical dominance within the academic publishing landscape (Larivière et al. 2015).

Yet, over recent decades, the scholarly publishing landscape has shifted dramatically towards FP models, leading to the dominance of a few publishers such as Springer, Reed-Elsevier, Wiley-Blackwell, and Taylor & Francis over an increasing share of high-impact journals (Posada and Chen 2018). For example, the five largest FP publishers accounted for 20% of publications in the 1970s, a figure that rose to 53% in 2013 (Larivière et al. 2015). This shift is closely tied to the still-growing reliance on journal-level metrics, particularly the impact factor (IF). Originally designed as a measure of citation frequency, the IF has rapidly been treated as a shorthand for journal excellence and prestige (Larivière and Sugimoto 2019). This dynamic potentially feeds a self-reinforcing cycle: high IFs attract more submissions, which generate greater publication volume and citations, further increasing visibility and IFs (figure 1).

The increasing prevalence of FP journals carries two major systemic consequences. First, it imposes rising financial burdens on institutions and researchers through rising subscription and article processing fees (Racimo et al. 2022, Kendall 2024). Second, it risks prioritizing financial gain over equitable knowledge dissemination (Bergstrom 2001, Bergstrom and Bergstrom 2004), challenging the public-good ethos of science (Ruggiero 2023, Haustein et al. 2024). For instance, the portfolio of Nature-branded journals (published by the FP publisher Springer Nature) has grown from a single flagship journal in 2009 to 34 journals in 2024 (McGill 2024), illustrating the accelerating commercialization of scientific publishing. This dynamic risks turning science into a “tragedy of the commons,” where collective benefits of open, equitable knowledge production are undermined by individual or institutional incentives (Ostrom 1990, Smaldino and McElreath 2016). The academic landscape is obviously more nuanced than a simple FP versus NP dichotomy. Some FP journals reinvest a substantial share of their revenues into academic activities such as research support and dissemination, earning the “academia-friendly” (AF) label (Galtier et al. 2026) (“for-profit academia-friendly” or “shared-profit” in the following). Nevertheless, these non-strictly FP journals face important system-wide disadvantages: they are fewer in number and therefore less visible in the academic publishing landscape than FP only journals (Larivière et al. 2015, McGill 2024), and their relative share has declined in recent years (McGill 2024).

## Citation patterns across journals’ business models

**Figure 2.**
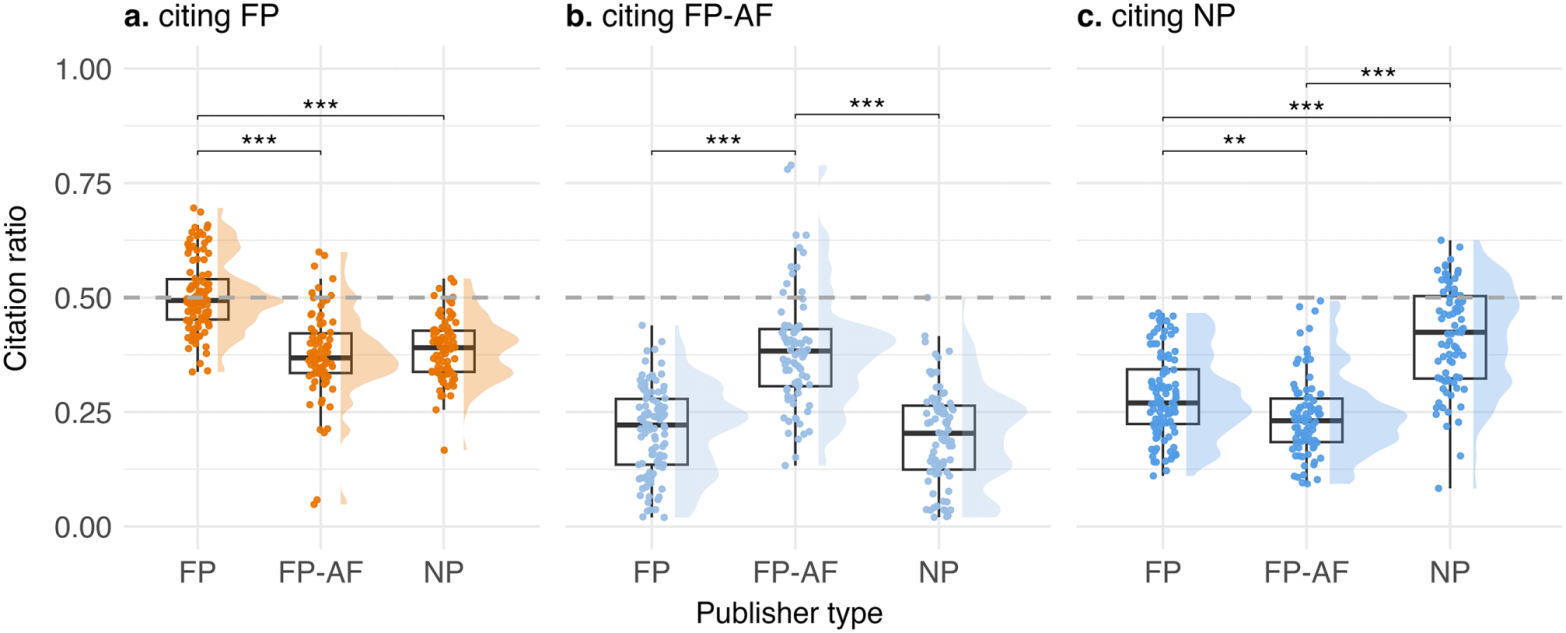
Citation pattern across journal business models. Proportion of citations in articles published in journals of different business models, shown separately for citations to (a) for-profit (FP), (b) for-profit academia-friendly (FP-AF), and (c) non-profit (NP) journals. Boxplots show medians and interquartile ranges; points and density curves show data distributions. Horizontal brackets indicate statistically significant differences in citation proportions between two publisher types based on Dunn tests with Bonferroni correction following significant Kruskal–Wallis tests (*p* < .001). Asterisks denote significance levels: *p < .05, **p < .01, and ***p < .001; non-significance is not indicated. Sample sizes were *n* = 106 (FP), *n* = 82 (FP-AF), and *n* = 82 (NP) journals.

A natural first step in addressing this imbalance is to examine how everyday scholarly practices reflect these asymmetries. Accordingly, we investigated whether citation patterns differ systematically across journals with different business models by analyzing cross-journal citations among FP, FP-AF, and NP journals. For clarity, we hereafter distinguish between “citations,” defined as countable acts of being cited; “references,” defined as the specific articles selected and listed by authors; and “citation practices,” defined as the norms shaping these choices and their associated biases. Using the field of ecology and evolution as a case study, we analyzed reference lists of more than 70,000 articles (published in 2023) across 270 journals across the three business types (box 1). We found that citation practices strongly cluster by journal business model: FP journals preferentially cite other FP journals, FP-AF journals cite FP-AF venues, and NP journals favor other NPs (figure 2; Dunn tests, *p* < .001). This clustering may partly reflect disciplinary proximity, as journals within similar research domains—often associated with specific business models—are more likely to cite one another. Alternatively, it may reflect the author’s tendency to cite references from the same journal as a strategy to increase the chances of publication acceptance (Wilhite and Fong 2012). This does not change the main conclusion but weakens the effect sizes, especially for FP journals (see Supplemental material S5). Although our analysis focused on DAFNEE-listed journals (Galtier et al. 2026), which are specific to ecology and evolution, similar structural imbalances likely exist in other scientific fields.

Even though citation practices are siloed across all journal business models, the citation bias is stronger for the FP journals, whose numerical dominance (Larivière et al. 2015, McGill 2024) in the academic publishing landscape creates a mechanistic advantage in citation accumulation and visibility (e.g., NP journals represented less than 10% of journals in 2021, all disciplines included [McGill 2024]). As a result, FP journals tend to receive more citations in absolute terms, which translates into higher IF. This visibility advantage may contribute to a self-reinforcing bias in citation practices toward FP journals (figure 1), if researchers cite the most accessible and prestigious literature rather than necessarily the most appropriate. Such a trend suggests that, without corrective efforts, AF journals (shared-profit and NP journals) risk further marginalization. Few flagship titles, such as Science (published by the American Association for the Advancement of Science and ranking among the highest IF journals in science [Thorp 2023]), may be able to retain their standing, whereas most journals risk being overshadowed by the growing landscape of FP journals with stronger citation performance and associated perceived prestige.

## Citation self-awareness

Researchers who wish to support a fairer academic publishing landscape can opt to exclusively publish in or review for shared- or non-profit journals. However, this strategy carries substantial risks, including limited publishing options, presumably lower visibility, and potential career setbacks. Albeit contested (Chapman et al. 2019, Larivière and Sugimoto 2019), IF remains a widely used proxy for academic excellence, so that boycotting purely FP journals creates disproportionate risks for early-career researchers as they are still pressured to publish in high-IF journals to advance their careers (Bonn and Pinxten 2021, Receveur et al. 2024). Amid debates over reforming evaluation metrics and funding models, one practical lever remains largely untapped: citation practices themselves.

Citations confer visibility, influence IF, and (should) signal scientific quality. Yet, researchers rarely reflect on how their own citation choices may be shaped by unconscious biases (such as journal visibility and prestige) that reinforce imbalances. We argue that raising awareness of how a journal business model can influence which research is most visible and frequently cited, combined with self-aware citation practices offer a lever to mitigate unintended citation bias and increase the visibility of shared- or non-profit journals. To support this approach, we introduce *fairpub* (available at https://github.com/FRBCesab/fairpub), an R package that enables authors to assess the balance of journal business models in their reference lists (e.g., based on a BibTeX file) and reflect on potential structural biases. At present, *fairpub* covers journals in ecology and evolution only, drawing on the DAFNEE database (Galtier et al. 2026); however, it is designed to be extended to journals from other scientific fields as comparable data become available.

What does this mean in practice? Importantly, when writing manuscripts, authors must follow the foundational ethical principle of scientific integrity: select the best citation to support their claims (Agarwal et al. 2023), irrespective of where it is published. However, this can be ambiguous, particularly for general background claims where multiple references may offer substantively equivalent support. In these situations, the awareness of citation bias becomes relevant. Scientific articles typically start with a review of the state of the art in the introduction that often includes general statements supported by well-known references (i.e., “The biodiversity crisis is accelerating worldwide,” “Biodiversity is important for ecosystem functioning”). Similar types of general or widely accepted claims also appear in the discussion or perspective sections. Where multiple references are scientifically equivalent, authors can prioritize those from shared- or non-profit journals. Such citation practices do not replace scientific rigour (Bonn and Pinxten 2021) but add awareness to reference choices already guided by content relevance. In doing so, it will help counterbalance the visibility advantage of FP journals, amplified by rising IF (figure 1), and mitigates resulting uneven accessibility between research published under different business models.

Box 1. **Characterizing current citation practices in ecology and evolution across journal business models.**

**Figure:**
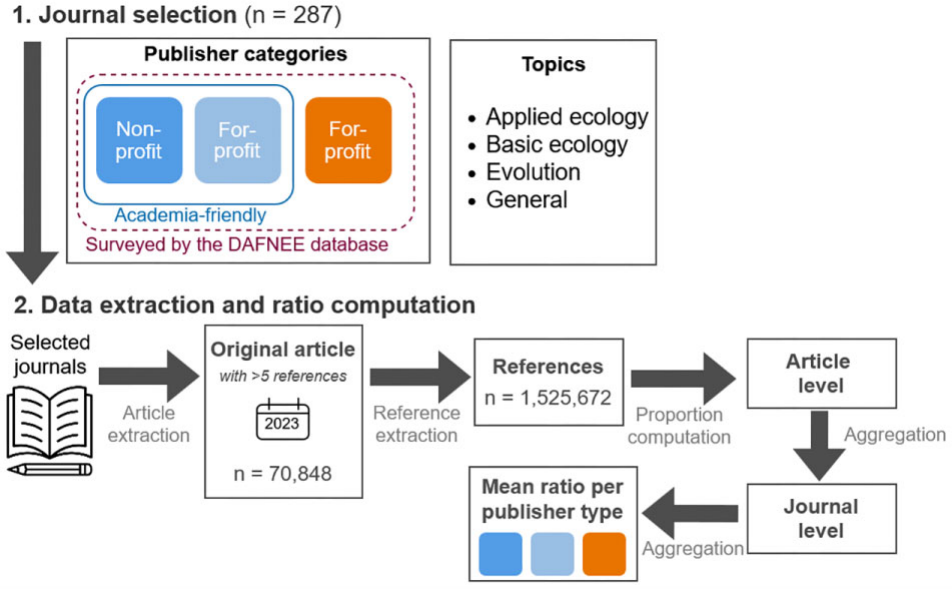

Our analysis uses the DAFNEE database (Galtier et al. 2026), which classifies journals based on their business model. We limited our analysis to journals within the thematic fields of “applied ecology,” “basic ecology,” “evolution,” and “general” to minimize disciplinary variation in citation practices. To represent the broader publishing landscape, we included FP-only journals from the list of journals surveyed by DAFNEE that matched our thematic fields (here, publisher information was obtained from journal websites). The final journal list comprised 287 journals: 93 NP, 83 FP-AF, and 111 FP journals.

Using OpenAlexR (Aria et al. 2024), we identified all articles published in 2023 by the selected journals (*n* = 130,324) and retrieved their cited references (*n* = 6,604,473). Each reference was classified based on the business model of its publishing journal into one of the three categories. Only references to journals in our selection were included in the further analysis. To reduce noise from atypical citation behavior and small sample sizes, we excluded publications with fewer than five references and removed special article types (e.g., comments, responses). This resulted in a final dataset of 70,848 original articles with 1,525,672 cited references (mean = 21.5 ± 18 SD references per article) from 270 journals (see Supplemental material S7). This filtering did not influence the general outcome (see Supplemental material S6).

For each article, we calculated the proportion of its references published in each of the three categories of journals (“citation ratio”). These values were aggregated at the journal level, and subsequently at the business model level, by computing unweighted mean proportions for each group. Additional methodological details are provided in the supplemental material.

## Looking forward

Change in citation practices must be approached with care. Following science ethics, authors cite the most appropriate publication to underpin their claims, irrespective of the venue in which it is published. Thus, excessively instrumentalizing citation choices risks to further devalue citation-based metrics, in line with Goodhart’s Law (“*When a measure becomes a target, it ceases to be a good measure*”). However, the academic publishing landscape is not ideal. Differences in journal visibility, accessibility, and prestige, particularly in a system where private profit plays a central role, suggest that citation practices are not shaped by relevance alone. In such a structurally asymmetrical landscape, citation choices are inevitably influenced by unconscious biases. Increasing citation self-awareness is necessary to help researchers recognize these biases, avoid being inadvertently guided by them, and contribute to a more balanced academic publishing landscape.

For this to be effective, researchers need to have access to transparent information on journal business models, yet this is currently not easily available. Although initiatives like the DAFNEE database (Galtier et al. 2026) and the *fairpub* package can provide valuable insights for journals in ecology and evolution, similar tools are largely missing in other disciplines. Ideally, a neutral regulatory body or institution should regularly evaluate and label journals according to their business model and reinvestment practices. Such tools and wider systemic transparency empower researchers to make informed, intentional choices without compromising scientific rigor. Until broader structural reforms—such as reducing reliance on IFs, increasing transparency in journal business models, and promoting more equitable publishing practices—are realized, self-aware citation practices provide a soft but powerful low-risk leverage to align everyday scholarly choices with broader goals of equity and sustainability in the scientific publishing ecosystem.

## Supplementary Material

biag028_Supplemental_File

## Data Availability

All data and code needed to evaluate the conclusions in the paper are present in the corresponding git repository: https://github.com/FRBCesab/ethical-citations.
