# Supplementary material for: Citation self-awareness for a fairer academic publishing landscape
Source: Bioscience. 2026 Apr 9;76(7):582–6. doi: 10.1093/biosci/biag028 (PMC13377635; doi:10.1093/biosci/biag028)
Supplement: biag028_Supplemental_File [file biag028_supplemental_file.pdf]

Supplementary Material

Materials and methods - Details

To characterize the landscape of citations in ecology and evolution, we retrieved all papers published in a selection of academic journals (see S7) for the year 2023. From each of these published articles, we retrieved all cited references to compute the proportion of references of articles published in for-profit (FP), for-profit academia-friendly (FP-AF) and non-profit (NP) journals. Finally, we compared these proportions between journals with distinct publishing models.

Tools and selection of journal publishing model data

We used the DAFNEE (Database of Academia Friendly jourNals in Ecology and Evolution, https://dafnee.isem-evolution.fr/) database to obtain a list of academic journals and their associated business models. The DAFNEE initiative represents an initial effort to centralise information regarding the business models of journals in the fields of ecology and evolution. As of July 1st 2025, a total of 1,169 journals have been surveyed. To be included in the database, on top of criteria such as peer-review and international scope, journals need to be 'academia-friendly', *i.e.* a criteria based on the existence of a visible partnership with an academic institution or a non-profit organization. For the >611 journals that were classified as academia-friendly, DAFNEE also informs on whether a journal publisher operates on a non-profit basis (NP), allocates part of its profits to science by supporting or partnering with an academic institution (university press), or is fully for-profit (FP-AF). For our analysis, the university press category was merged into the NP category because we considered that the benefit would be used to serve the scientific community through the university. From these 611 academia-friendly journals, we kept 172 journals in the fields of 'applied ecology', 'basic ecology', 'evolution' and 'general'. Among the remaining international scope journals that were surveyed but deemed non-academia-friendly (and therefore not listed in the DAFNEE database), we selected those relevant to the same categories to represent the whole variety of journal landscape (n=128). The publisher type of these journals was not provided by DAFNEE and was manually obtained based on information on the journal's website. The few journals identified as NP generally belonged to publishers linked to the scientific community (n=13) and were thus integrated into the category of NP journals.

To retrieve articles and their cited references, we used the OpenAlex bibliographic database (Priem et al. 2022). OpenAlex is an open-access database of scholarly publications and a reliable alternative to the proprietary bibliometric databases (such as Web of Science and Scopus, Culbert *et al.* 2025). Furthermore, it is well suited for reproducible research: we used the R package openalexR (Aria *et al.* 2024) to query their API and retrieve article metadata.

Article and reference extraction

Among the 300 initial academic journals we sampled, only 287 were indexed in the OpenAlex database. We limited our analysis to 2023 and extracted metadata of all articles published in these 287 academic journals during that year. Only 270 academic journals

published articles in 2023 with a total of 130,324 articles (median = 103 articles per journal, mean = 475.64 +/- 1848.25 SD). Finally, from each article, we extracted all cited references (n = 6,604,473, median = 48, mean = 50.68 +/- 33.77 SD per article).

Citation ratio computation

We matched each cited reference with its journal's publishing model (FP, FP-AF, NP). As we did only have this information available for our selected journals list, the number of articles with covered references (*i.e.*, references citing works that were among our list of journals) decreased to 110,988 and the number of cited references decreased to 1,633,350. In order to reduce biases due to a low number of cited references and to remove special article types (*i.e.*, comment, response), we excluded articles with less than 5 cited references. After applying this filter, our data comprised 70,848 articles with 1,525,672 cited references (mean = 21.53 +/- 18.01 SD references per article) from 270 journals (S1). Including articles citing <5 references did not affect the overall finding (S6).

	For-profit	For-profit academia friendly	Non-profit	Total
**Journals**	106	82	82	270
**Articles**	43,285	10,661	16,902	70,848
**Cited references**	901,020	281,281	343,371	1,525,672

S1. *Number of journals, articles, and cited references after filtering used to compute citation ratio per publisher type.*

For each article, we calculated the proportion of the three categories of cited references, considering only those for which publisher type information was available (as described above). Then we calculated the journal-level mean proportions by averaging ratios of all articles per journal. The distributions of these citation ratios are presented in Fig. 2 in the main manuscript. Finally, we averaged these journal-level values within publisher type (S2).

Differences in citation practices between journals following distinct publisher types were tested for significance by Wilcoxon tests, followed by Dunn Test. P-values were adjusted for multiple comparisons following the 'Bonferroni' method (Dunn, 1961).

		Citation ratio		
		**For-profit**	**For-profit academia-friendly**	**Non-profit**
**Publisher type**	**For-profit**	0.50 +/- 0.08	0.21 +/- 0.09	0.28 +/- 0.09
	**For-profit academia friendly**	0.38 +/- 0.09	0.38 +/- 0.12	0.24 +/- 0.09
	**Non-profit**	0.39 +/- 0.07	0.20 +/- 0.11	0.41 +/- 0.11

S2. *Mean proportion and standard deviation of for-profit, for-profit academia-friendly and non-profit references in journals published by the three types of publishers.*

Sensitivity of results

Sensitivity towards self-citation

We repeated the calculation and aggregation after removing self-citing references, *i.e.* references published in the same journal as the original article. This reduced the number of articles and cited references (S3) to be considered and the effect size (S4, S5).

	**For-profit**	**For-profit academia friendly**	**Non-profit**	**Total**
**Journals**	106	82	82	270
**Articles**	38,015	8,620	14,786	61,421
**Cited references**	777,685	236,572	289,979	1,304,236

S3. *Number of journals, articles, and cited references after filtering used to compute citation ratio per publisher type after removing self-citation*

		Citation ratio		
		**For-profit**	**For-profit academia-friendly**	**Non-profit**
**Publisher type**	**For-profit**	0.45 +/- 0.07	0.24 +/- 0.10	0.32 +/- 0.11
	**For-profit academia friendly**	0.43 +/- 0.09	0.28 +/- 0.12	0.28 +/- 0.12
	**Non-profit**	0.43 +/- 0.07	0.22 +/- 0.12	0.34 +/- 0.11

S4. *Mean proportion and standard deviation of for-profit, for-profit academia-friendly and non-profit references in journals published by the three types of publishers after removing self-citation.*

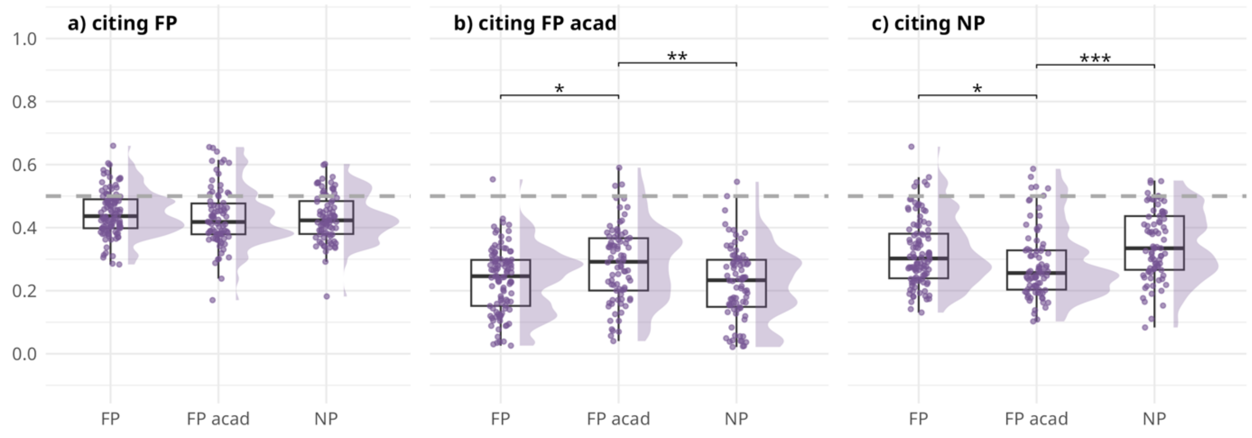

S5: *Proportion of citations towards articles published in for-profit (a) for-profit academic-friendly (b) and non-profit journals (c) among the same three publisher types after removing self-citation, i.e. articles citing the journal they are publishing in.*

<u>Sensitivity towards filtering steps</u>

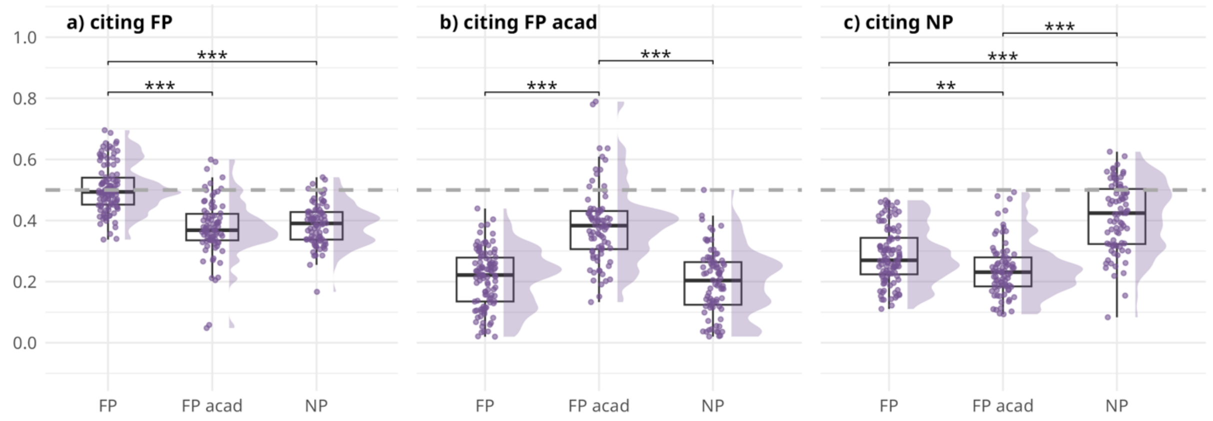

S6: *Proportion of citations towards articles published in for-profit (a) for-profit academic-friendly (b) and non-profit journals (c) among the same three publisher types including articles with <5 references.*

Code availability

The code to reproduce data extraction and analysis is available on GitHub: https://github.com/frbcesab/ethical-citations

***S7**. List of 270 journals included in the analysis that published articles in 2023 and their classification into business type categories*

For-Profit	For-Profit (academia-friendly)	Non-Profit
acta ethologica	African Journal of Ecology	Acta Amazonica
Acta Oecologica	African Journal of Range and Forage Science	African Journal of Wildlife Research
Applied Soil Ecology	Agronomy for Sustainable Development	Animal Biodiversity and Conservation
Aquaculture Environment Interactions	AMBIO	Annual Review of Ecology Evolution and Systematics
Aquatic Botany	Animal Behaviour	Antarctic Science
Aquatic Conservation Marine and Freshwater Ecosystems	Animal Conservation	Aquatic Ecosystem Health & Management
Aquatic Ecology	Annals of Forest Science	Behavioral Ecology
Behavioral Ecology and Sociobiology	Annals of the New York Academy of Sciences	Biological Journal of the Linnean Society
Behaviour	Applied Vegetation Science	Biology Letters
Biodiversity and Conservation	Aquatic Living Resources	Biology Open
Biodiversity Data Journal	Archives of Sexual Behavior	BioScience
Biological Conservation	Austral Ecology	Bird Conservation International
Biological Invasions	Basic and Applied Ecology	Bulletin of Entomological Research
BMC Ecology and Evolution	Behavior Genetics	Bulletin of Marine Science
BMC Zoology	Biological reviews/Biological reviews of the Cambridge Philosophical Society	Canadian Journal of Fisheries and Aquatic Sciences
Communications Biology	Biotropica	Canadian Journal of Forest Research
Crustaceana	Bulletin of the Ecological Society of America	Cities and the Environment
Current Biology	Chelonian Conservation and Biology	Cold Spring Harbor Perspectives in Biology
Current Opinion in Insect Science	Cladistics	Comptes Rendus Biologies
Diversity	Coastal Management	Conservation Physiology
Diversity and Distributions	Conservation and Society	Ecology and Society
Ecological Complexity	Conservation Biology	eLife
Ecological Indicators	Conservation Letters	Environmental Conservation
Ecological Informatics	Conservation Science and Practice	European Journal of Taxonomy
Ecological Modelling	Ecography	Evolution
Ecology and Evolution	Ecological Applications	Evolution Letters
Ecology Of Freshwater Fish	Ecological Monographs	Evolutionary Journal of the Linnean Society
Ecosystems	Ecological Research	Freshwater Science
Endangered Species Research	Ecological Solutions and Evidence	Frontiers of Biogeography
Environmental Biology of Fishes	Ecology	Gayana
Environmental DNA	Ecology Letters	ICES Journal of Marine Science
Environmental Microbiology	Ecoscience	Ideas in Ecology and Evolution

Environmental Microbiology Reports	Ecosphere	Integrative and Comparative Biology
European Journal of Wildlife Research	Environmental Science and Pollution Research	Interface Focus
EvoDevo	Environmental Toxicology and Chemistry	International Journal of Plant Sciences
Evolution & Development	Estuarine Coastal and Shelf Science	International Journal of Wildland Fire
Evolutionary Applications	European Journal of Soil Science	Invasive Plant Science and Management
Evolutionary Biology	Evolutionary Systematics	Invertebrate Systematics
Evolutionary Ecology	Frontiers in Ecology and the Environment	Journal of Coastal Research
Fire	Functional Ecology	Journal of Economic Entomology
Food Webs	Hormones and Behavior	Journal of Experimental Biology
Forest Ecology and Management	International Journal of Remote Sensing	Journal of Pollination Ecology
Forests	Journal of Applied Ecology	Journal of the National Museum (Prague) Natural History Series
Freshwater Biology	Journal of Arid Land	Journal of Threatened Taxa
Frontiers in Ecology and Evolution	Journal of Chemical Ecology	Journal of Tropical Ecology
Frontiers in Marine Science	Journal of Coastal Conservation	Marine and Freshwater Research
Frontiers in Microbiology	Journal of Ecohydraulics	Mediterranean Marine Science
Global Change Biology	Journal of Ecology	National Science Review
Global Ecology and Biogeography	Journal of Evolutionary Biology	Natural Areas Journal
Global Ecology and Conservation	Journal of Limnology	New Zealand Journal of Ecology
Hydrobiologia	Journal of Mammalian Evolution	Northwestern Naturalist
IAWA Journal	Journal of Sustainable Agriculture and Environment	Novitates Caribaea
Insects	Journal of Systematics and Evolution	Open Biology
International Journal of Fisheries and Aquatic Studies	Journal of the Marine Biological Association of the United Kingdom	Oryx
iScience	Journal of Vegetation Science	Pacific Conservation Biology
Journal for Nature Conservation	Journal of Wildlife Management	Parasitology
Journal of Applied Entomology	Knowledge and Management of Aquatic Ecosystems	Peer Community In Ecology
Journal of Arid Environments	Landscape Ecology	Peer Community in Evolutionary Biology
Journal of Biogeography	Management of Biological Invasions	Peer Community In Forest and Wood Sciences
Journal of Environmental Management	Marine Ecology Progress Series	Peer Community In Registered Reports
Journal of Experimental Marine Biology and Ecology	NeoBiota	Philosophical Transactions of the Royal Society B Biological Sciences

Journal of Forestry Research
Journal of Land Use Science
Journal of Pest Science
Journal of Sea Research
Journal of Theoretical Biology
Journal of Zoological Systematics & Evolutionary Research
Land
Limnologica
Mammal Research
Mammalia
Marine Biodiversity
Marine Biology
Marine Biology Research
Microbial Ecology
Molecular Ecology
Molecular Ecology Resources
Molecular Phylogenetics and Evolution
Movement Ecology
Mycorrhiza
Nature
Nature Communications
Nature Ecology & Evolution
Nature Plants
Nature Sustainability
Neotropical Biodiversity
Nova Hedwigia
Oecologia
One Earth
Open Journal of Ecology
Parasitology Research
PeerJ
Perspectives in Plant Ecology Evolution and Systematics
Phytotaxa
Plant Systematics and Evolution
Review of Palaeobotany and Palynology
Reviews in Fish Biology and Fisheries
Scientific Data
Scientific Reports
Studies on Neotropical Fauna and Environment
New Phytologist
Oikos
Open Research Europe
Organisms Diversity & Evolution
Pedosphere
People and Nature
Personality and Individual Differences
Perspectives in Ecology and Conservation
Population Ecology
Remote Sensing in Ecology and Conservation
Restoration Ecology
Taxon
Tropical Ecology
Urban Ecosystems
Vegetation Classification and Survey
Weed Research
Wellcome Open Research
Wetlands
Wildlife Biology
Wildlife Society Bulletin
Zoosystematics and Evolution
Philosophy Theory and Practice in Biology
Phytobiomes Journal
Plant Ecology and Evolution
PLoS Biology
PLoS ONE
PNAS Nexus
Polish Journal of Ecology
Proceedings of the National Academy of Sciences
Proceedings of the Royal Society B Biological Sciences
Records of the Australian Museum
Royal Society Open Science
Science
Science Advances
Scientia Marina
Silva Balcanica
Sociobiology
Systematic Biology
The American Naturalist
The Bulletin of zoological nomenclature
The Coleopterists Bulletin
Web Ecology
Wildlife Research
Zoological Journal of the Linnean Society
Zoosystema

Thalassas An International
Journal of Marine Sciences
The Botanical Review
The Science of Nature
Theoretical Ecology
Theoretical Population Biology
Trends in Ecology & Evolution
Tropical Conservation Science

References

Aria, M, Le T, Cuccurullo C, Belfiore A & Choe J (2024) openalexR: An R-tool for collecting bibliometric data from OpenAlex. The R Journal, 15(4), 167-180. https://doi.org/10.32614/RJ-2023-089

Culbert JH, Hobert A, Jahn N *et al.* (2025) Reference coverage analysis of OpenAlex compared to Web of Science and Scopus. Scientometrics 130, 2475–2492. https://doi.org/10.1007/s11192-025-05293-3

Dunn, OJ. (1961) Multiple comparisons among means. J. Am. Stat. Assoc. 1961:56(293):52–64. https://doi.org/10.1080/01621459.1961.10482090

Priem J, Piwowar H & Orr R (2022) OpenAlex: A fully-open index of scholarly works, authors, venues, institutions, and concepts. ArXiv. https://arxiv.org/abs/2205.01833.
